# Collagen-grafted porous HDPE/PEAA scaffolds for bone reconstruction

**DOI:** 10.1186/s40824-016-0071-5

**Published:** 2016-07-27

**Authors:** Chang-Shik Kim, Kyung-Hye Jung, Hun Kim, Chan-Bong Kim, Inn-Kyu Kang

**Affiliations:** 1 Department of Polymer Science and Engineering, Kyungpook National University, Daegu, 702-701 South Korea; 2 Department of Advanced Materials and Chemical Engineering, Catholic University of Daegu, Kyungsan, South Korea; 3 Jeil Medical Corporation, Seoul, South Korea

**Keywords:** High-density polyethylene, Porous scaffolds, Collagen, Osteoblasts, Differentiation

## Abstract

After tumor resection, bone reconstruction such as skull base reconstruction using interconnected porous structure is absolutely necessary. In this study, porous scaffolds for bone reconstruction were prepared using heat-pressing and salt-leaching methods. High-density polyethylene (HDPE) and poly(ethylene-co-acrylic acid) (PEAA) were chosen as the polymer composites for producing a porous scaffold of high mechanical strength and having high reactivity with biomaterials such as collagen, respectively. The porous structure was observed through surface images, and its intrusion volume and porosity were measured. Owing to the carboxylic acids on PEAA, collagen was successfully grafted onto the porous HDPE/PEAA scaffold, which was confirmed by FT-IR spectroscopy and electron spectroscopy for chemical analysis. Osteoblasts were cultured on the collagen-grafted porous scaffold, and their adhesion, proliferation, and differentiation were investigated. The high viability and growth of the osteoblasts suggest that the collagen-grafted porous HDPE/PEAA is a promising scaffold material for bone generation.

## Background

For bone reconstruction such as skull base reconstruction after tumor resection, an interconnected porous structure is critical to mimicking the bone extracellular matrix [1–8]. The pore size, porosity, and pore interconnectivity of porous bone scaffolds determine their performance in functions such as cell attachment and nutrient diffusion, which enhances soft tissue and bone ingrowth and eventually resistance to infection or deformation. Moreover, mechanical stability is mandatory for the mechanical support that is required during the repair and regeneration of damaged or degenerated bone [7, 9]. Porous scaffolds for biomedical applications have been successfully fabricated via the sol-gel process [10], salt-leaching method [8, 11–13], electrospinning [14–17], and microsphere-sintering technique [18, 19]. However, the lack of mechanical strength of the porous materials can cause instability of the pore structures and hence limit their biomedical applications, and thus the choice of scaffold material is crucial.

The performance of porous scaffolds can be optimized by controlling their surface chemistry, because the interface between the porous scaffolds and cells determines the cellular behavior, such as cell adhesion, spreading, and proliferation [6]. Collagen is the main organic component of bones, and is hence a promising candidate material for the surface modification of porous scaffolds by promoting cell attachment and chemotactic responses [20].

High-density polyethylene (HDPE) shows excellent mechanical properties, and it has been widely used as an implant material for bone reconstruction [18, 21, 22]. Medpor® (Porex Technologies Co., USA) is one such porous HDPE scaffold for bone tissue engineering, used as an alloplastic material for craniofacial reconstruction [23, 24]. However, HDPE is inert and hydrophobic, and exhibits poor reactivity with biomaterials such as collagen. Several efforts have been made to improve the reactivity of PE for biomedical applications. The grafting of acrylic acid onto the PE film was conducted to improve protein immobilization and cell seeding [25]. It was also reported that plasma treatment effectively provides HDPE with a hydrophilic surface, which results in better reactivity with bioactive molecules [26]. The carboxylic acid groups of poly(ethylene-co-acrylic acid) (PEAA) make it an outstanding candidate to support the reactivity with collagen. Besides this, PEAA is mechanically stable, owing to the strong hydrogen bonds in its carboxylic acid groups, which can be effective crosslinkers between polymer chains.

In this study, the composite of HDPE and PEAA was chosen as scaffold material for cranial reconstruction owing to the high mechanical stability of HDPE and the high reactivity of PEAA with collagen. Before collagen grafting, the porous structure was prepared using a salt-leaching method, which can provide the proper pore size and high porosity. Osteoblast cells were then cultured on the collagen-grafted porous HDPE/PEAA scaffold, and the cell adhesion, proliferation, and differentiation were measured to investigate their bone tissue compatibility. Porous scaffolds of HDPE and HDPE/PEAA without collagen grafting were also fabricated and studied as controls.

## Methods

### Fabrication of collagen-grafted porous scaffolds

Porous HDPE/PEAA scaffolds were fabricated by using a salt-leaching method^10^. HDPE (Mw 85,000, Mn 13,500; Korea Petrochemical Industrial Co., Korea) and PEAA (acrylic acid 20 wt%; Sigma-Aldrich Co., USA) beads (w/w = 3:1) were mixed with sodium chloride (HDPE/PEAA:NaCl = 1:9) with a particle size of 200–500 μm, using a melt mixing machine (Brabender, Plasti-Corder Co.) at 160 °C. Then, the mixture was cast in a circular mold (diameter 13 mm, thickness 1.3 mm) using a heat press machine (Yoochang Co., Korea). The resulting HDPE/PEAA/NaCl composite was immersed in distilled water to leach out the NaCl, leaving pores in the composite. The salt-free porous HDPE/PEAA was washed with distilled water and air dried.

For obtaining high reactivity between the scaffold and collagen, L-lysine was grafted onto the scaffold surface to improve the affinity of the carboxyl groups to the amine groups in collagen. Before the L-lysine grafting, the carboxylic groups on the HDPE/PEAA scaffold were activated by immersing the scaffold into a 1-ethyl-3-(3-dimethylaminopropyl) carbodiimide (0.25 wt%; Sigma-Aldrich Co., USA) and N-hydroxysuccinimide (0.25 wt%; Sigma-Aldrich Co., USA) aqueous solution for 6 h at room temperature. Afterwards, it was immersed in 3 wt% L-lysine aqueous solution with gentle stirring. The carboxyl groups of L-lysine, attached to the scaffold surface, were also activated by this same method. Collagen-grafted HDPE/PEAA (HDPE/PEAA/Col) was produced by immersing the HDPE/PEAA scaffold in 3 wt% collagen solution (in distilled water containing acetic acid, pH 4.3) for 6 h with gentle stirring, and then it was washed with distilled water and dried.

### Characterization of the scaffolds

The surface morphology of the porous HDPE, HDPE/PEAA, and HDPE/PEAA/Col scaffolds was observed under a field emission scanning electron microscope (FE-SEM S4300; Hitachi, Japan) after sputter-coating with platinum. The chemical bonds and elemental composition were characterized by Fourier transform infrared (FT-IR; Mattson, Galaxy 7020A) spectroscopy and electron spectroscopy for chemical analysis (ESCA; ESCA LAB VIG microtech, Mt 500/1, and so forth, East Grinstead, UK), respectively.

Tensile properties were measured via a universal testing machine (Instron, model 4465) with a Zwick Roell tensile tester equipped with a 1 kgf load cell, at 25 °C with an extension speed of 10 mm/min. The tensile strength and Young’s modulus measure of each sample were calculated from the averages of 10 specimens.

The porosity of the porous scaffolds was determined by using a mercury intrusion porosimeter (AutoPore IV 9520; Micromeritics Co., USA). The advancing and retreating contact angles of mercury were taken to be 140° and the surface tension was taken as 0.480 N/m (480 dynes/cm).

### Cell behavior

Cell behavior was observed by culturing osteoblast cells (5 × 10^4^ cells/mL; MC3T3-E1, ATCC) on the scaffolds, at 37 °C in a humidified atmosphere with 5 % CO_2_, in Dulbecco’s modified Eagle’s medium (Gibco, USA) supplemented with 10 % fetal bovine serum (Gibco, USA) and 1 % penicillin G-streptomycin (Gibco, USA). After both 1 and 2 days of incubation, calcein-AM (1 mM in dimethyl sulfoxide) and propidium iodide (1.5 mM in distilled water) solutions were added and the scaffolds were left standing for 15 min. The fluorescence images were visualized with a confocal laser scanning microscope (CLSM, Carl Zeiss, LSM 700, Germany).

To evaluate the cytoskeletal organization of cells on the porous scaffolds, double staining was performed. After 3 days of incubating the cell solution with the scaffold samples, the cells were fixed with 4 % paraformaldehyde in PBS and permeabilized with 0.1 % Triton X-100 in PBS for 15 min. The samples were then incubated for 30 min in a PBS containing 1 % bovine serum albumin, followed by the addition of tetramethylrhodamine-5-isothiocyanate (TRITC)-conjugated phalloidin (Millipore, Cat. No. 90228). After 1 h, the samples were incubated with 4,6-diamidino-2-phenylindole (DAPI) (Millipore, Cat. No. 90229) for 5 min. The fluorescence images were taken with a confocal laser scanning microscope (CLSM 700).

The cell viability and proliferation on the porous scaffolds were evaluated using the 3-(4,5-dimethylthiazol-2-yl)-2,5-diphenyltetrazolium bromide (MTT) assay and enzyme-linked immunosorbent assay (ELISA). For the MTT assay, the scaffold samples were immersed in 50 μL of MTT solution (5 mg/mL in PBS) for 4 h. After removing the solution, the water-insoluble formazan product was dissolved in 0.04 N HCl-isopropanol in the dark. ELISA was performed using 5-bromo-2-deoxyuridine (BrdU), which is incorporated during DNA synthesis in the cells. The BrdU ELISA was conducted according to the manufacturer’s instructions (Roche Molecular Biochemicals, Germany). The absorbance was measured at 570 nm, using a kinetic microplate reader (EL × 800; Bio-T Instruments, Inc., Highland Park, USA).

Cell differentiation was tested by several cell staining methods, using alizarin red S, von Kossa, and alkaline phosphatase (ALP) staining. The osteoblast cells (5 × 10^4^ cells/mL) were cultured for 15 days on the three porous scaffolds and then fixed using 10 % formaldehyde. For alizarin red S staining, the samples were treated with an alizarin red S solution and incubated for 20 min. For the von Kossa assay, the fixed samples were treated with 5 % AgNO_3_ solution for 20 min under ultraviolet radiation, followed by the addition of 5 % Na_2_S_2_O_3_ solution for 5 min. ALP staining was done by a standard procedure according to the manufacturer’s instructions (Alkaline phosphatase, Leukocyte, Procedure No. 86; Sigma-Aldrich, USA), using an alkaline dye mixture (1 mL of sodium nitrate, 1 mL of FBB-alkaline solution, 1 mL of naphthol AS-BI alkaline solution, and 1 mL of deionized water) and a neutral red buffered solution for counterstaining [27]. The digital images of the stained cultures were obtained with a digital camera (Canon A2000 IS, Japan) and an optical microscope (Carl Zeiss, Germany).

### Data analysis

The results are displayed as the mean ± standard deviation. The statistical significance of differences between the scaffolds was determined by a Student’s two-tailed *t* test. Scheffe’s method was used for multiple comparison tests at a level of 95 %.

## Results and discussion

### Pore structure

**Fig. 1:**
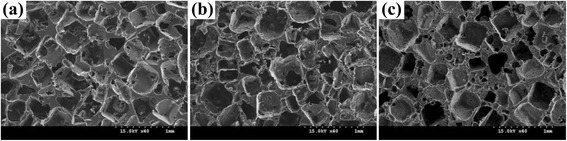
Surface morphologies of the porous HDPE (**a**), HDPE/PEAA (**b**) and HDPE/PEAA/Col (**c**) scaffolds

The surface morphology of the porous HDPE, HDPE/PEAA, and HDPE/PEAA/Col scaffolds was observed by scanning electron microscopy. As shown in Fig. 1, interconnected pores were successfully formed in the scaffolds, and their pore sizes ranged between several microns and a few hundred microns. It is also seen that the collagen-grafted scaffold in Fig. 1(c) had slightly smaller pores than those without collagen grafting in Fig. 1(a) and (b).

The intrusion volume and porosity were measured to investigate the change of pore size by the scaffold materials and collagen grafting, and the results are shown in Table 1. The porosity of the HDPE/PEAA scaffold was similar to that of HDPE, which was approximately 65 %. However, when collagen was introduced to the surface of the HDPE/PEAA scaffold the porosity decreased by 5 %, likely due to the high molecular weight of collagen.

**Table 1:** Intrusion volume and porosity of the porous HDPE, HDPE/PEAA and HDPE/PEAA/Col scaffolds

Substrate	Intrusion volume (mL/g)	Porosity(%)
HDPE	2.08	65.21
HDPE/PEAA	2.31	66.75
HDPE/PEAA/Collagen	1.88	59.28

Standard deviation is within 10 %

The pore characteristics are also key factors that affect the performance of porous scaffolds in bone reconstruction because the pore size and porosity of scaffolds affect the diffusion of nutrients and osteoblast cell attachment, migration, proliferation, and differentiation, which are vital for bone formation. Additionally, a porous surface is known to drive mechanical stability at the interface between the implant materials and the surrounding tissue [28]. Even though there is disagreement about the optimum pore size of porous scaffolds, it is generally agreed upon that the pore size and porosity play essential roles in their compatibility to cells such as osteoblasts, and pores of a few hundred microns are highly required [3–5, 8]. Therefore, on the basis of the results of Fig. 1 and Table 1, it can be concluded that the pore size of the HDPE-based scaffolds prepared by the salt-leaching method is appropriate for porous bone scaffolds.

### Surface chemistry

**Fig. 2:**
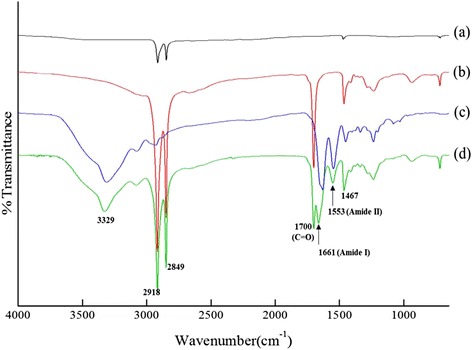
ATR-FTIR spectra of (a) HDPE (—), (b) HDPE/PEAA (—), (c) Collagen (—), and (d) HDPE/PEAA/Col (—)

FT-IR spectra of the HDPE, HDPE/PEAA, and HDPE/PEAA/Col scaffolds and of collagen are shown in Fig. 2. Both the HDPE and HDPE/PEAA spectra exhibited bands at 2849 and 2918 cm^−1^, assigned to hydrocarbons (CH, CH_2_). For the HDPE/PEAA scaffold (Fig. 2b), the vibrational band at 1700 cm^−1^ based on C = O was observed, but it did not appear for the HDPE scaffold (Fig. 2a), which proves that PEAA was well incorporated into the HDPE/PEAA scaffold. It is also seen that the HDPE/PEAA/Col scaffold (Fig. 2d) displayed the characteristic collagen peaks at 1661 and 1553 cm^−1^, assigned to the stretching vibration of the carbonyl group (C = O) within amide I (–**CO**NH–) and the coupling of N-H bending and C-N stretching of amide II (–CO**NH**–), respectively.

Collagen grafting on the HDPE/PEAA scaffold was further confirmed by ESCA, and the elemental compositions of the HDPE, HDPE/PEAA, and HDPE/PEAA/Col scaffolds are shown in Table 2. The atomic percentage of nitrogen was significantly increased on the surface of the HDPE/PEAA scaffold modified with L-lysine and subsequently with collagen. According to the FT-IR spectra and ESCA results, it can be confirmed that collagen grafting was successfully conducted on the porous HDPE/PEAA scaffold.

**Table 2:** Chemical composition of porous scaffolds calculated from their survey scan spectra

Substrate	Atomic %					
	C 1 s	O 1 s	N 1 s	Si 2p	CI 2p	Na 1 s
HDPE	93.5	5.3	<0.1	1.2	-	-
HDPE/PEAA	83.2	11.8	1.0	2.4	0.3	0.5
HDPE/PEAA/Collagen	81.8	11.1	5.3	1.2	0.5	0.1

### Tensile properties

**Fig. 3:**
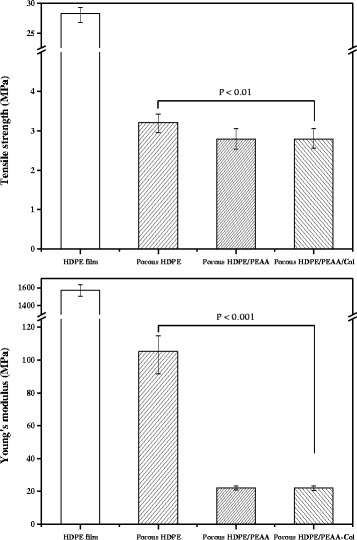
Tensile properties of the porous HDPE, HDPE/PEAA scaffolds

Figure 3 represents the tensile strength and Young’s modulus measures of the porous HDPE, HDPE/PEAA, and HDPE/PEAA/Col scaffolds. The porous HDPE scaffold showed higher strength and modulus values, owing to the high mechanical stability of HDPE. When PEAA was incorporated into the HDPE scaffold, its Young’s modulus measure decreased significantly, while the tensile strength was slightly lowered. It is also shown that grafting collagen on the scaffolds does not affect their tensile properties. PEAA is widely used as a compatibilizer for polymer blends or composites because of its functionality. Its segment of acrylic acid provides unique properties, such as polarity, crosslink ability, and adhesion to polar substrates, as well as low softening and melting points [29]. Kim et al. reported the addition of PEAA to polyethylene terephthalate/HDPE blends, which effectively improved their mechanical properties such as flexural yield strain and impact strength [30]. PEAA was also reported as a compatibilizer of polylactic acid/recycled low-density polyethylene blends, enhancing the tensile properties of the composites [31].

### Cell viability and proliferation

**Fig. 4:**
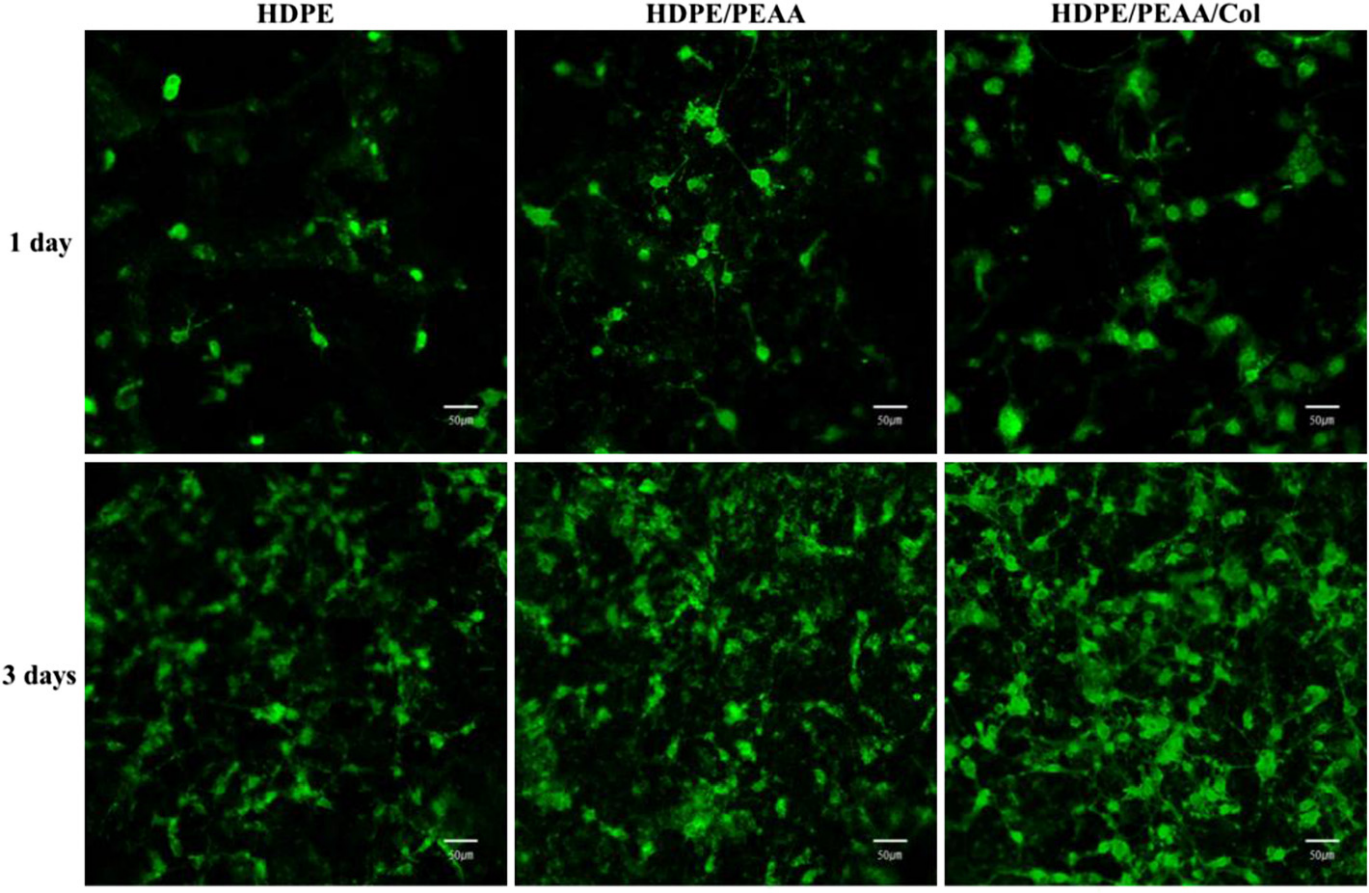
Confocal laser scanning microscope images of calcein-AM dye-stained osteoblast cells cultured on the porous HDPE, HDPE/PEAA, and HDPE/PEAA/Col scaffolds

Cell behaviors on the HDPE, HDPE/PEAA, and HDPE/PEAA/Col scaffolds were investigated using several methods to examine their biocompatibility. First, the adhesion and cytotoxic effects of the three scaffolds were observed by using cell staining after a 1-day and 3-day incubation period. Figure 4 shows the morphologies of osteoblast cells on the surface of the scaffolds. Calcein-AM, a highly lipophilic dye that can easily penetrate the cell membrane, interacts with cytosolic esterase in viable cells to result in green fluorescence. All the cells in Fig. 4 exhibited strong green fluorescence, indicating the good viability of osteoblast cells. On the porous HDPE scaffold, only a few cells had adhered, and their growth appeared to be somewhat slow (Fig. 4a and d). On the other hand, the HDPE/PEAA scaffold (Fig. 4b and e) displayed slightly better cell adhesion and cell spreading, and these properties were further enhanced when collagen was introduced to the surface of the HDPE/PEAA scaffold (Fig. 4c and f).

**Fig. 5:**
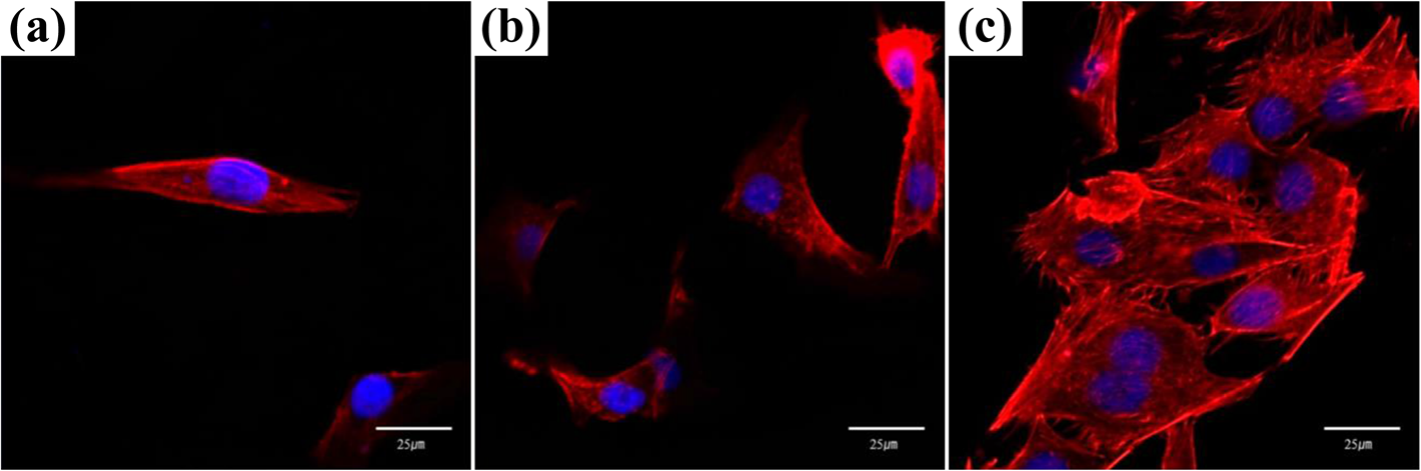
Confocal laser scanning micrographs (actin in red, nucleus in blue) of osteoblast cells cultured on the porous HDPE (**a**), HDPE/PEAA (**b**), and HDPE/PEAA/Col (**c**) scaffolds

The nucleus and actin of the osteoblast cells cultured on the three different scaffolds were observed by double staining to examine the cytoskeleton organization. As shown in Fig. 5, actin was stained with TRITC (red), whereas the nucleus was stained with DAPI (blue). It appeared that the cells cultured on the HDPE scaffold (Fig. 5a) expressed actin filaments slightly with a small number of cells. However, the cytoskeletons of the cells on the HDPE/PEAA scaffold (Fig. 5b) seemed more organized. The HDPE/PEAA/Col scaffold (Fig. 5c) showed a large number of cells cultured on the substrate that were clearly organized with stretched actin and stress fibers.

**Fig. 6:**
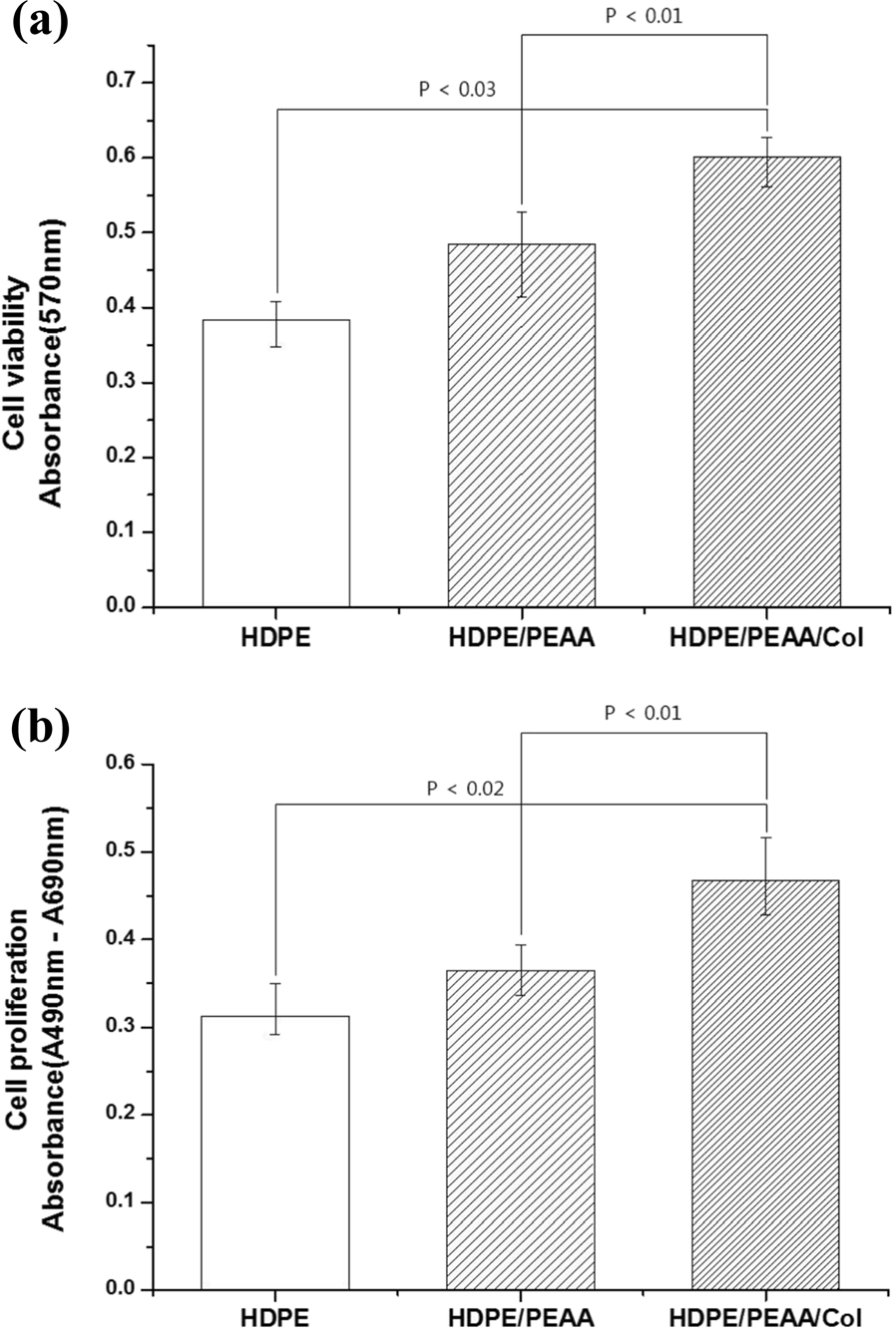
MTT (**a**) and BrdU (**b**) assays of osteoblast cells cultured on the porous HDPE, HDPE/PEAA, and HDPE/PEAA/Col scaffolds

Figure 6 shows the MTT and BrdU assay results after a 3-day incubation of osteoblast cells on the three scaffolds. The HDPE/PEAA/Col scaffold showed significantly higher cell viability and proliferation (*p* < 0.03 for MTT and *p* < 0.02 for BrdU assays) than the HDPE and HDPE/PEAA scaffolds, suggesting that collagen plays an important role in cell growth and metabolism. Not only were a large number of osteoblasts alive, but they also proliferated actively on the collagen-containing biocompatible scaffold. Collagen has been mainly used to improve biocompatibility via surface modification for biomedical applications [32–34]. It was reported that collagen grafting successfully promoted cell proliferation by cell growth and cell division on both organic and inorganic materials.

### Cell differentiation

**Fig. 7:**
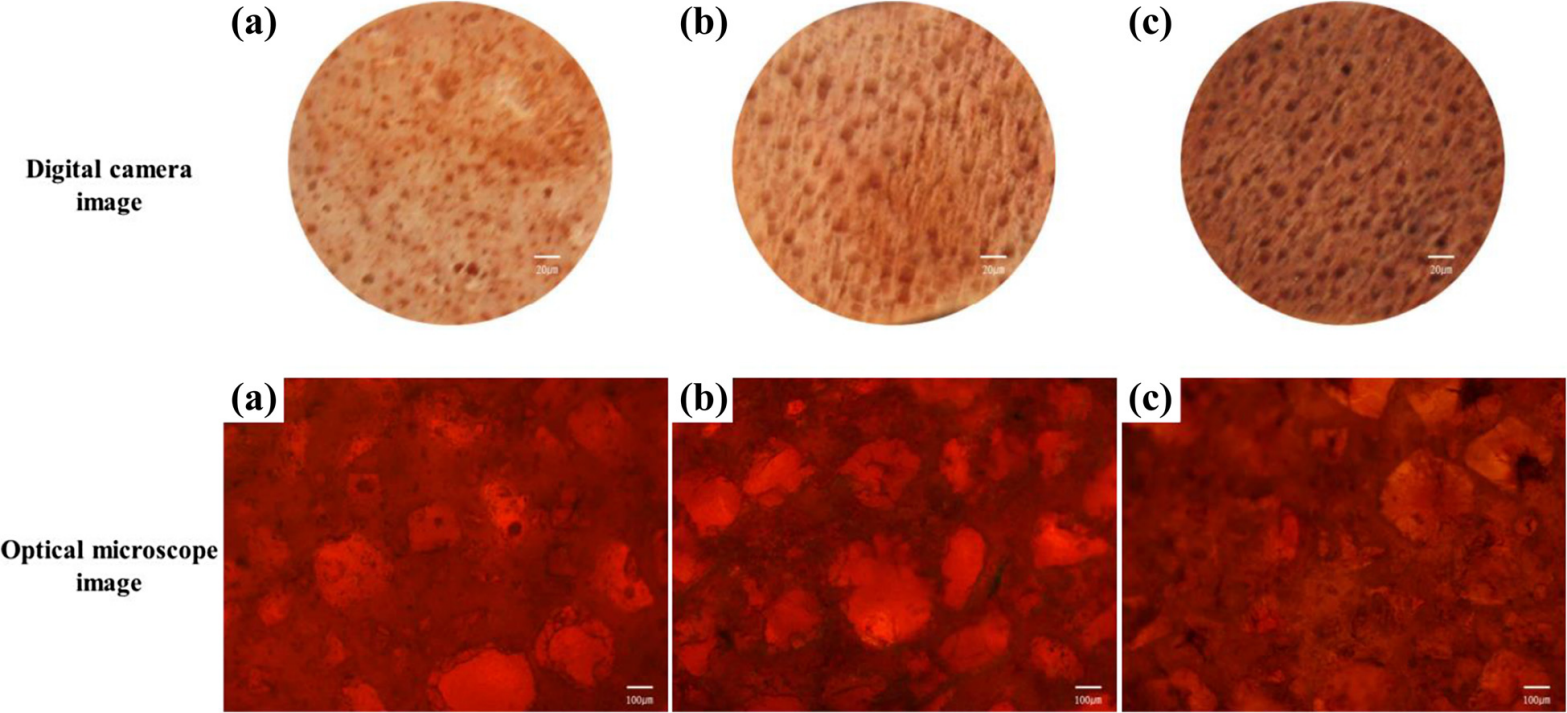
Alizarin red S staining of osteoblast cells cultured on the porous HDPE (**a**), HDPE/PEAA (**b**), and HDPE/PEAA/Col (**c**) scaffolds

Osteoblast cell differentiation is one of the most important parameters for confirming the osteogenesis of osteoblast cells. Alizarin red S, von Kossa, and ALP staining methods have been frequently utilized to characterize the interface between calcified bone tissue and the implant surface [35–37]. For alizarin red S staining, the calcification area in the cells is stained red from the formation of a calcium/alizarin red S complex. Figure 7 shows the result of alizarin red S staining of osteoblast cells on the three porous scaffolds. It can be seen that osteoblasts on the HDPE, HDPE/PEAA, and HDPE/PEAA/Col scaffold were stained in red, with the HDPE/PEAA/Col scaffold showing the most intense dark red color, resulting from accelerated cell differentiation by collagen grafting.

**Fig. 8:**
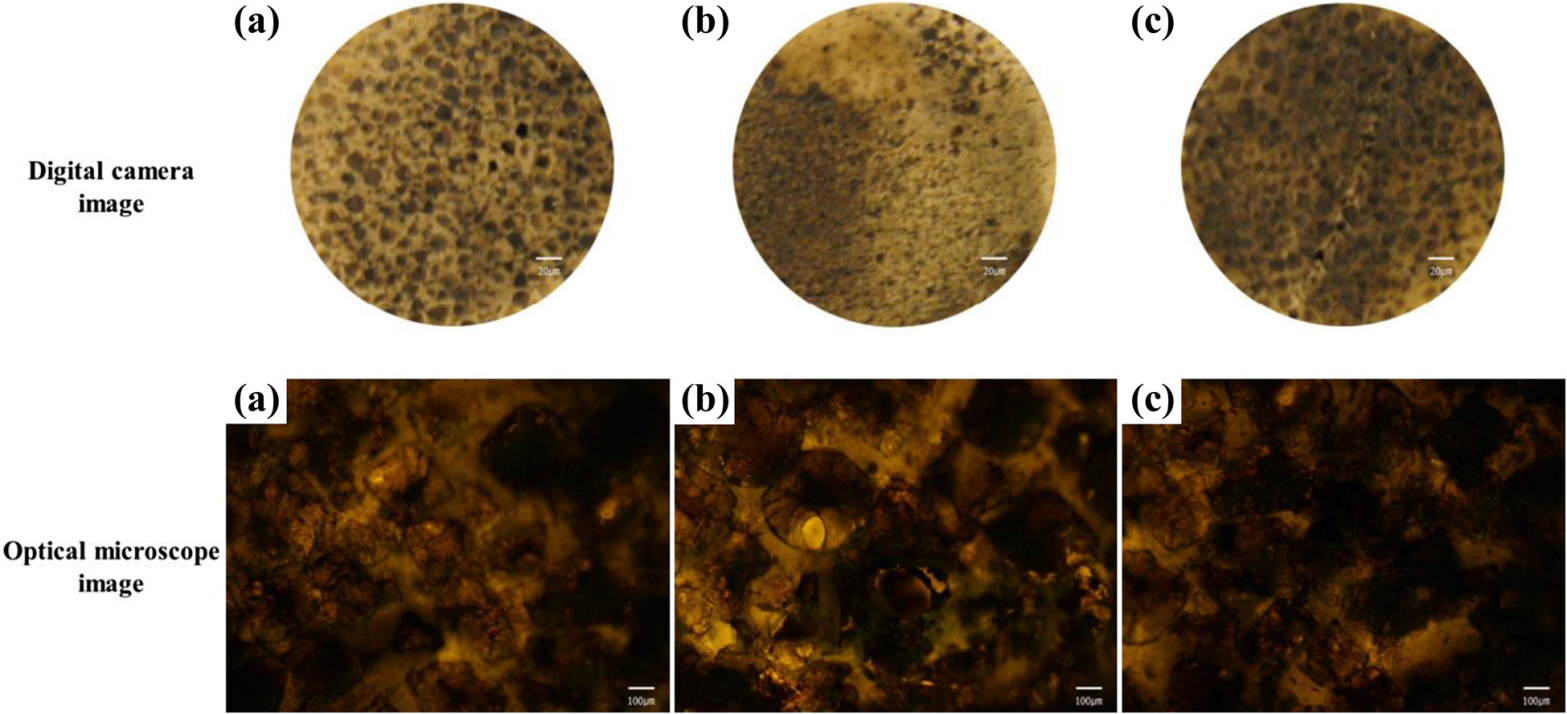
Von Kossa assay of osteoblast cells cultured on the porous HDPE (**a**), HDPE/PEAA (**b**), and HDPE/PEAA/Col (**c**) scaffolds

The von Kossa stain is also one of the ways to confirm mineralization in cell cultures by detecting phosphate in the calcification area, which is stained as a black spot. In Fig. 8, von Kossa staining images of osteoblast cells cultured on the porous scaffolds are displayed. Osteoblast cells on the HDPE/PEAA/Col scaffold showed the most intensive dark spots among the three scaffolds. It was therefore confirmed that collagen grafting is effective in triggering or accelerating osteoblast cell differentiation, which matches well with the alizarin red S staining results in Fig. 7. The HDPE/PEAA scaffold presented better cell differentiation (Figs. 7b and 8b) than the HDPE scaffold (Figs. 7a and 8a).

**Fig. 9:**
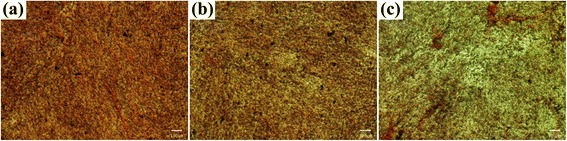
ALP activity staining of osteoblast cells cultured on the porous HDPE (**a**), HDPE/PEAA (**b**), and HDPE/PEAA/Col (**c**) scaffolds

The differentiation of osteoblast cells was further proven by the synthesis of ALP in the cells, which appears as a blue spot (Fig. 9). ALP is an enzyme produced by osteoblast activity such as bone generation. Therefore, the amount of ALP synthesized can represent the vitality of osteoblast cells. The amount of ALP synthesized by the cells cultured on the HDPE/PEAA/Col scaffold (Fig. 9c) was higher than those on the HDPE and HDPE/PEAA scaffolds (Fig. 9a and b, respectively). Thus, the osteoblasts on the collagen-grafted scaffold had active ALP synthesis, indicating the scaffold’s potential for bone generation applications.

## Conclusions

For bone reconstruction, porous scaffolds were fabricated using HDPE/PEAA composites via a salt-leaching method. The surface of the porous HDPE/PEAA scaffold was modified using collagen to enhance bone tissue compatibility. The surface modification was confirmed via FT-IR spectroscopy and ESCA by detecting the nitrogen component in collagen. It was shown that the pore size and porosity are suitable for osteoblast attachment, as confirmed by the surface images and porosity results. The cell viability and proliferation were measured by MTT and BrdU assays, with results showing that the collagen-grafted HDPE/PEAA surface is favorable for the adhesion and proliferation of osteoblast cells. Furthermore, cell differentiation was studied using several staining methods, where it was seen that osteoblasts on the collagen-grafted scaffold have outstanding differentiation. It is concluded that collagen grafting on the porous HDPE/PEAA scaffold effectively improves its biocompatibility and potential use as a bone scaffold.
